# Use of percutaneous image-guided coaxial core-needle biopsy for diagnosis of intraabdominal lymphoma

**DOI:** 10.1002/cam4.224

**Published:** 2014-07-08

**Authors:** Ikuo Shimizu, Yoichi Okazaki, Wataru Takeda, Takehiko Kirihara, Keijiro Sato, Yuko Fujikawa, Toshimitsu Ueki, Yuki Hiroshima, Masahiko Sumi, Mayumi Ueno, Naoaki Ichikawa, Hikaru Kobayashi

**Affiliations:** 1 Department of Hematology, Nagano Red Cross HospitalNagano-city, Nagano, Japan; 2 Center for Medical Education, Shinshu University School of MedicineMatsumoto, Nagano, Japan; 3 First Department of Radiology, Nagano Red Cross HospitalNagano-city, Nagano, Japan

**Keywords:** Diagnosis, immunophenotype, malignant lymphoma, needle biopsy, sensitivity and specificity

## Abstract

Although pathological diagnosis is essential for managing malignant lymphoma, intraabdominal lesions are generally difficult to approach due to the invasiveness of abdominal surgery. Here, we report the use of percutaneous image-guided coaxial core-needle biopsy (CNB) to obtain intraabdominal specimens for diagnosing intraabdominal lymphomas, which typically requires histopathological and immunohistochemical evaluation. We retrospectively reviewed consecutive cases involving computed tomography (CT)- or ultrasonography (US)-guided CNB to obtain pathological specimens for intraabdominal lesions from 1999 to 2011. Liver, spleen, kidney, and inguinal node biopsies were excluded. We compared CNBs with laparotomic biopsies. A total of 66 CNBs were performed for 59 patients (32 males, 27 females; median age, 63.5), including second or third repeat procedures. Overall diagnostic rate was 88.5%. None of the patients required additional surgical biopsies. Notably, the median interval between recognition of an intraabdominal mass and biopsy was only 1 day. Forty-five procedures were performed for hematological malignancies. Adequate specimens were obtained for histopathological diagnosis in 86% of cases. Flow cytometry detected lymphoma cells in 79.5% of cases. Twelve patients (nine males, three females; median age, 60) were eligible for surgical biopsy. While every postoperative course was satisfactory, median duration from lesion recognition to therapy initiation for lymphoma cases was significantly shorter for CNB than for surgical biopsy (14 vs. 35 days). While one-fourth of the patients were not eligible for the procedures, CNB is safe and highly effective for diagnosis of intraabdominal lymphomas. This method significantly improves sampling and potentially helps attain immunohistological distinction, allowing for more timely therapy initiation.

## Introduction

Intraabdominal lesions are some of the most prevalent types of non-Hodgkin's lymphoma, especially in Japan 1–3, and often present as solitary abdominal masses. Thus, sampling of pathological specimens poses a challenge. Although considered the gold standard, surgical biopsy is invasive, and tracheal intubation and general anesthesia are not free of complications. Image-guided core-needle biopsy (CNB) has emerged as an alternative procedure, and is now established as a method for organ biopsies that is less invasive than surgery and allows for prompt pretreatment evaluations. While needle biopsy is a reliable method, some reports are available for intraabdominal lymph nodes sampling 4–9. Reports on needle biopsy include those concerning solitary mass lesions, as well as organ lesions. Moreover, no report has directly compared needle biopsy with surgical biopsy. While immunophenotypic and genetic characteristics play an increasingly important role in the diagnosis of lymphoid malignancies 2, few reports have assessed the diagnostic rates of flow cytometry (FCM) and chromosomal studies combined with needle sampling 10,11.

Coaxial fine-needle biopsy is expected to improve diagnostic rates, as it makes repeat sampling possible and allows for immunochemical evaluation. In our hospital, image-guided coaxial CNB has been commonly performed by a well-trained certified radiologist in order to sample tissues of intraabdominal lesions including lymph nodes. In this study, we retrospectively reviewed CNB cases to address the following questions: (1) What is the accuracy of diagnosis based on results of CNB in patients with malignant lymphoma? and (2) Does CNB shorten the duration of pretreatment evaluation of patients with lymphoma compared to surgical biopsy?

## Patients and Methods

During an initial evaluation of patients with intraabdominal lesions or lymph nodes at our hospital between April 1999 and March 2011, 66 image-guided CNB procedures were performed for 59 patients by an experienced certified radiologist (Y. O.). We considered CNB a first-line procedure. Twenty patients underwent surgical biopsies for lesions which were not percutaneously approachable.

Intraabdominal or retroperitoneal mass lesions, which were considered lymph nodes, were the target sites in this study. Percutaneous biopsies of organs such as the liver, kidney, spleen, and inguinal lymph node were excluded from evaluation. Therapeutic surgeries were also excluded.

All CNBs were performed under image control (computed tomography [CT] or ultrasonography [US]) using a 15- or 18-gauge Quick-Core Biopsy Needle (Cook Co., Bloomington, IN).

Biopsy materials were fixed in 10% buffered formalin for histological evaluations or directly submitted for immunochemical or cytogenetic evaluation. The sections were then subjected to immunohistochemistry and stained with hematoxylin–eosin as needed. In addition, flow cytometric analyses and chromosomal studies by G-banding were performed by SRL (Tokyo, Japan). Evaluations by fluorescent in situ hybridization or T-cell receptor rearrangement were also combined as needed. Histological results were based on the interpretation of biopsy materials prepared according to standard techniques used in the Department of Pathology of our hospital by experts, in combination with flow cytometric analyses and cytogenetic evaluation.

CT-guided CNB procedures at our hospital are as follows. First, CT images are obtained to determine the puncture site. Then, an outer (guide) needle is inserted under laser guidance and local anesthesia is administered. While keeping the guide needle in the lesion with a safety margin, tissue samples are obtained with a cutting needle. Finally, follow-up CT scans are performed to confirm no bleeding or accidental injuries. Most patients are usually admitted for a night to monitor adverse events.

Correlations between the two groups were examined by chi-square analysis, the one-sided Fisher exact test, and the Student's *t-*test. *P* < 0.05 was considered statistically significant. All the analyses were performed using PASW statistical software, version 18.0.

This retrospective study was approved by the Institutional Review Board of Nagano Red Cross Hospital.

## Results

**Figure 1:**
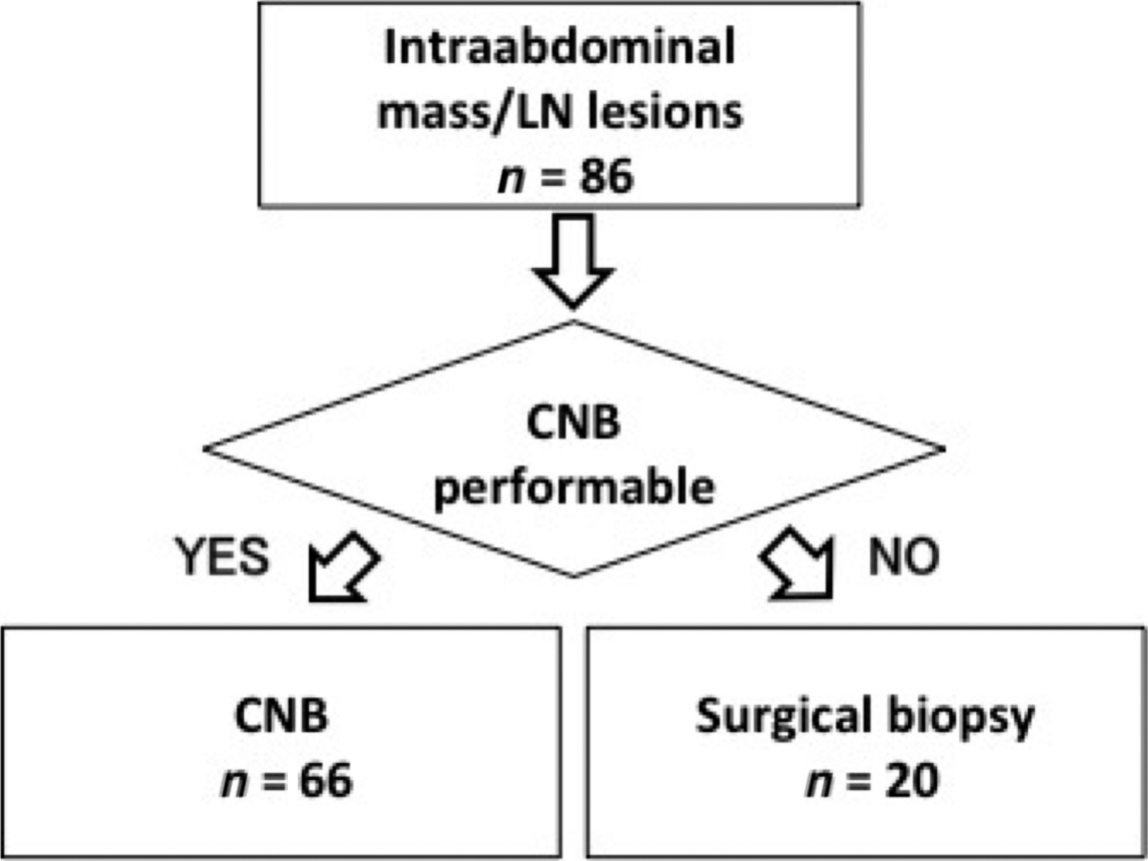
Study design. LN, lymph node; CNB, core-needle biopsy.

There were a total of 66 CNBs performed for 59 patients (CNB group). The procedure was repeated twice in four patients and three times in one patient because specimen was insufficient for evaluation in four procedures, and one relapsed patient required another biopsy for rediagnosing. No other patients were required repeated biopsy or classical lymphoid biopsy. Twenty surgical biopsies were performed for 20 patients (surgery group), as shown in Figure1. Table 1 shows the characteristics of patients in both groups. All elderly patients aged 75 years and older underwent CNB. Most intraabdominal lesions located dorsally and near vertebrae were subjected to CNB, while deeper lesions were sampled surgically.

**Table 1:** Characteristics of needle biopsy and surgical biopsy groups

	CNB	Surgical biopsy
Procedures/patients	66/59^1^	20/20
Males/females	32/27	15/5
Median age (range)	63.5 (24–85)	60 (42–72)
>75 years old	11	0
Imaging modalities	CT 51 US 15	–
Surgical procedures	–	Laparotomy 15 Laparoscopic surgery 2
Sampling sites	Paraaortic LN 24 Mesenteric LN 13 Paravertebral mass 9 Retroperitoneum mass 8 Pelvic mass 6 Adrenal gland 4 Splenic LN 1 Parapancreatic mass 1	Mesenteric LN 9 Paraaortic LN 5 Retroperitoneal mass 3 Hepatic LN 1 Ext. iliac LN 1 Omentum mass 1

CNB, core-needle biopsy; LN, lymph node; CT, computed tomography; US, ultrasonography.

1 Needle biopsy was performed repeatedly seven times for five patients (six inaccurate procedures and one relapse).

**Figure 2:**
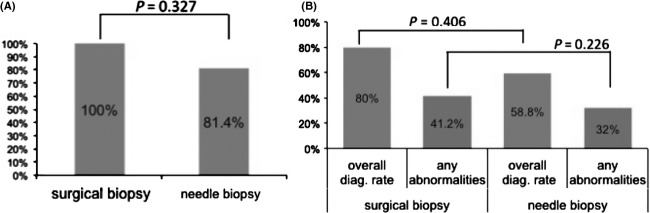
Flow cytometric (FCM) analysis and chromosomal diagnostic rates of lymphoma cases. (A) Immunochemical studies by FCM. There was no significant difference in diagnostic rates between the groups. (B) Chromosomal studies by G-band. Overall diagnostic rates and ratios for detecting any chromosomal abnormalities related to lymphoid malignancies were not significantly different between the groups.

The median number of guided needle samplings in one CNB procedure was three (range, 1–8), and only one puncture was required for obtaining enough samples in 44% of procedures. Table 2 shows the final pathological diagnoses for all patients. Two patients who underwent CNB were diagnosed solely based on clinical and cytological evaluations. Fifty-six patients in the CNB group and 15 in the surgery group had malignant disease, and the most common diagnosis was malignant lymphoma. Aggressive lymphomas were more frequently detected in the CNB group. On the other hand, more patients in the surgical group were diagnosed with indolent lymphomas. All seven cases of unknown primary malignancy in the CNB group were finally diagnosed through further evaluation other than direct sampling. The overall diagnostic rate of CNB was 88.5% including first and repeated biopsies; sensitivity and specificity were 85.9% and 100%, respectively. Diagnostic rates for malignant lymphoma were not significantly different between CNB and surgical biopsy groups (CNB 86% vs. surgery 100%, *P* = 0.836). Of note, there was no significant difference in diagnostic rates between the two imaging modalities (CT 85.4% vs. US 92.3%, *P* = 0.66). None of the patients evaluated in this study had serious complications, including 11 elderly patients aged over 75 years who underwent CNB. Immunotypical analysis by FCM, which was obtained in 81.4% (35/43) in CNB, revealed no significant difference in diagnostic rates between the groups (100% vs. 81.4%, *P* = 0.327), as shown in Figure2A. In chromosomal studies by G-band method (Fig.2B), overall diagnostic rates of CNB and surgery, 58.8% (20/34) and 80% (8/10) (*P* = 0.406) and ratios for detecting any chromosomal abnormalities related to lymphoid malignancies, 32% (12/38) and 41.3% (7/17) (*P* = 0.226), did not differ significantly between the groups. With regard to complications, neither CNB nor surgery cases experienced any types of complications such as bleeding, perforation, or infection, among others.

**Table 2:** Pathological diagnosis

	CNB (*n* = 59)	Surgical biopsy (*n* = 20)
Lymphoma	39 DLBCL 16, FL 12, Hodgkin 3, PTCL-NOS 3, SLL 3, Burkitt 1, ENKL 1	13 FL 8, DLBCL 4, Hodgkin 1
Other malignancy	17 Rhabdomyosarcoma 4, Endometrial cancer 1, Gastric cancer 1, Thymic cancer 1, Esophageal cancer 1, Neurofibromatosis 1, Pancreatic 1, Unknown origin 7	2 Prostate cancer 1, Cystadenocarcinoma 1
Benign condition	3 Tuberculosis 1, Reactive 2	5 IgG4-related 1, Sarcoidosis 1, Reactive 3

CNB, core-needle biopsy; DLBCL, diffuse large B-cell lymphoma; FL, follicular lymphoma; PTCL-NOS, peripheral T-cell lymphoma, not otherwise specified; SLL, small lymphocytic lymphoma; ENKL, extranodal NK/T-cell lymphoma, nasal type.

**Figure 3:**
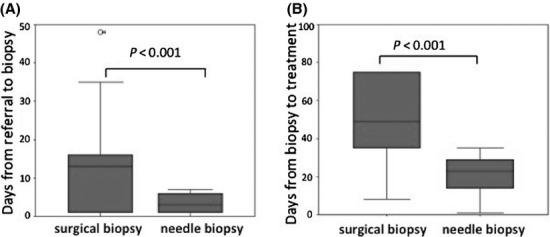
Median days required for pretreatment evaluation. (A) Days from referral to biopsy. (B) Days from biopsy to treatment excluding those under “watchful wait” cases and those who refused treatment. Median days for evaluation were significantly shortened in CNB group.

We also compared the median duration required for pretreatment evaluation, as shown in Figure3. Median duration from referral to biopsy was significantly shortened in the CNB group at 1 day (0–7) in comparison to the surgery group at 16 days (0–48) (*P* < 0.001). Excluding patients under “watchful wait” and those who refused treatment, the median duration from biopsy to treatment was also significantly shorter in the CNB group at 14 days (1–35) than the surgery group at 35 days (3–58) (*P* < 0.001).

## Discussion

Needle biopsies of abdominal mass lesions have been used to establish a malignant diagnosis in patients with clinical suspicion. While some have reported that the sensitivity of fine-needle aspiration (FNA) cytology combined with FCM was greater than that of CNB 10,11, the feasibility is controversial; another report states that CNB provides more accurate subtyping of some tumors than does FNA 6. In this study, we were able to reach a final diagnosis with CNB samples including minimal exception based on FNA.

Some studies have reported on the accuracy of CNB for diagnosing abdominal masses 6,9,10. Silverman et al. reported no false-positive CNB results compared with surgical correlative data 12. No previous report has evaluated CNB and surgical biopsy simultaneously; it is especially difficult to compare the diagnostic accuracy between the two. In this study, however, the diagnostic rate of CNB was not significantly inferior compared with surgical biopsy, and no change in final diagnosis was observed during the clinical course following biopsy. Given the futility of over-repeated biopsy in most cases, we believe that these results are sufficient to confirm the diagnostic accuracy of CNB.

Hashimoto et al. 13 previously reported that almost 90% of lymphomas are associated with chromosomal abnormalities. In this study, however, abnormalities were observed less frequently with CNB, suggesting that some technical limitations may exist for this procedure. Notably, surgical biopsy also showed a lower diagnostic rate for chromosomal abnormalities, with no significant difference compared with that of CNB. While CNB was not inferior to surgical biopsy for evaluating chromosomal abnormalities of lymphoma specimens, further improvements are needed.

We used large (15- or 18-) gauge needles for CNBs in order to obtain tissues. Interestingly, finer needles were used in a study that suggested diagnostic superiority of FNA in comparison to CNB 11. One study found no difference in outcome between each of the three needle sizes, although the sample size might be considered small (13–33 patients) 14. However, several others have reported CNB as a generally safe method 6,8,14. Although no difference was previously reported between coaxial and noncoaxial methods for liver or kidney biopsies 15, they experienced seven (0.9%) major complications including one death. In contrast, there were no major complications associated with CNB performed under the Cook core-needle system in this study. A previous study reported that needle biopsy is a safe and effective procedure for elderly people 16. Consistent with this, none of the patients in the CNB group, including elderly patients aged over 75 years, experienced any adverse events in this study. Since all elderly patients were eligible for CNB, we were unable to compare the efficacy among this cohort. Although more studies will be needed to further evaluate the safety of CNB, we consider this procedure a suitable option for elderly patients.

Recently, several studies have reported on the feasibility of endoscopic ultrasonography (EUS)-FNA for the diagnosis of intraabdominal malignancies, including malignant lymphomas 17–21. Adequate specimens were obtained in more than 90% of cases, with high sensitivity and specificity 20,21. Interestingly, it is also indicated that classification of lymphoma subtypes was possible in 80% of cases 21. Yet, there are several limitations with EUS-FNA worth noting. First, evidences of EUS-FNA is mainly based on aspiration, not tissue biopsy. According to the latest WHO classification, diagnosis is based on pathological examination 2; therefore, cytodiagnosis by aspiration biopsy would be insufficient. Second, lymphoid malignancy, especially of NK/T-cell lineage, is often difficult to evaluate with immunophenotyping alone 22. The report from Japan provides a good example of this; all but one of the 12 malignant lymphomas evaluated successfully were of a B-cell lineage (diffuse large B-cell lymphoma, *n* = 6; follicular lymphoma [FL], *n* = 5; Hodgkin lymphoma, *n* = 1) 21. Another report showed that EUS-FNA failed to establish a specific pathological diagnosis other than lineage determination in one-third of cases evaluated 20.

The most common indolent lymphoma is FL, and requires grading when pathologically determined, often transforming during the clinical course. It occurs at a rate of approximately 3% per year for the first 10 years 23, and an autopsy series reports transformation rates up to 70% 24. Evaluations based on aspiration specimens are virtually impossible, even in combination with FCM. On the other hand, as we presented, CNB allows for pathological and immunophenotypical evaluations. Therefore, in order to establish a diagnosis of intraabdominal lymphadenopathy in clinically suspected lymphoma, we believe that pathological evaluation with CNB should be performed as much as possible, especially when NK/T-cell lineage lymphoid malignancies or indolent B-cell lymphomas are suspected. The possibility to evaluate the latest subclassification by CNBs is a further research question.

There are several limitations to this study. First, we did not collect data concerning specimen size or the number of viable cells obtained by CNBs. The threshold for evaluating accurately the lymph nodes in CNB is still not clear. According to the classification put out by the WHO, anything longer than 1.5 cm in length is adequate for bone marrow evaluation 2. Further details are required for clarification. Second, aggressive lymphomas were more easily identified with CNB, whereas indolent lymphomas were more easily identified by surgery. This distinction might be attributed to a possible selection bias in patients who presented with aggressive clinical courses due to difficulty indicating general surgery. However, the shortened duration required for evaluation, without decreasing diagnostic rates, is obviously beneficial in the clinical practice. The third limitation is that, although statistical analyses showed beneficial effects of CNB in this study, the study sample comprised a limited number of individuals from a single institution. In addition, since CNB procedures were performed by a single operator, the results could be biased. Similarly, potential advantage of CNBs for shortening the duration may depend on the regional factors for pathological diagnosis and the standard postoperative care in Japan, which usually requires long hospitalization. Multicenter data with larger sample sizes are required to confirm these results.

In conclusion, despite these limitations, CNB is highly useful for pathological diagnosis of abdominal lymphadenopathy in patients suspected of lymphoma. It allows for immunohistochemical evaluation by way of FCM, as well as chromosomal evaluation. In addition, the pretreatment duration is significantly shortened compared with surgery. Tissue sampling by CNB is thus considered adaptable for diagnosis of intraabdominal lesions.
